# Restoration of Genetic Code in Macular Mouse Fibroblasts via APOBEC1-Mediated RNA Editing

**DOI:** 10.3390/biom15010136

**Published:** 2025-01-16

**Authors:** Sonali Bhakta, Hiroko Kodama, Masakazu Mimaki, Toshifumi Tsukahara

**Affiliations:** 1 Bioscience, Biotechnology and Biomedical Engineering Research Area, Japan Advanced Institute of Science and Technology, Nomi 923-1211, Japan; sonali.dvm@gmail.com; 2 Department of Anatomy and Histology, Faculty of Veterinary Science, Bangladesh Agricultural University, Mymensingh 2202, Bangladesh; 3 General Medical Education and Research Center, Teikyo University School of Medicine, Tokyo 173-0003, Japan; 4 Department of Pediatrics, Teikyo University School of Medicine, Tokyo 173-0003, Japan; 5 GeCoRT Co., Ltd., Nishi-ku, Yokohama 220-0011, Japan

**Keywords:** RNA editing, macular mouse, fibroblast, APOBEC 1, MS2 system, guide RNA

## Abstract

RNA editing is a significant mechanism underlying genetic variation and protein molecule alteration; C-to-U RNA editing, specifically, is important in the regulation of mammalian genetic diversity. The ability to define and limit accesses of enzymatic machinery to avoid the modification of unintended targets is key to the success of RNA editing. Identification of the core component of the apoB RNA editing holoenzyme, APOBEC, and investigation into new candidate genes encoding other elements of the complex could reveal further details regarding APOBEC-mediated mRNA editing. Menkes disease is a recessive X-chromosome-linked hereditary syndrome in humans, caused by defective copper metabolism due to mutations in the *ATP7A* gene, which encodes a copper transport protein. Here, we generated plasmids encoding the MS2 system and the APOBEC1 deaminase domain and used a guide RNA with flanking MS2 sites to restore mutated *Atp7a* in fibroblasts from a macular mouse model of Menkes disease withs T>C mutation. Around 35% of the mutated C nucleotide (nt) was restored to U, demonstrating that our RNA editing system is reliable and has potential for therapeutic clinical application. RNA base editing via human RNA-guided cytidine deaminases is a potentially attractive approach for in vivo therapeutic application and provides opportunities for new developments in this field.

## 1. Introduction

Genome editing technologies, such as the clustered regularly interspaced short palindromic repeat (CRISPR) and CRISPR-associated proteins (Cas) systems, are anticipated to enable the development of innovative therapies for various disorders [1,2,3]. To modify a genome, these techniques must create site-specific double-strand DNA breaks (DSBs), which are then repaired by endogenous systems, including either the error-prone non-homologous end-joining pathway or the error-free homology-directed repair pathway [3,4,5]. In addition, many DNA editing methods [6,7,8] have been developed in recent years that can modify a particular DNA nucleotide (nt) in a genome using enzymes that deaminate adenosine or cytidine. These techniques are predicted to be less risky for gene therapy applications than traditional genome editing technologies, because they do not involve a DSB phase, which generates minor insertion or deletion alterations that bridge the break site [9,10,11,12,13,14,15,16,17].

RNA editing is another significant mechanism that can modify hereditary traits and allow elective protein modification through changes at the single-nt level. RNA editing can achieve nucleotide substitution by various genetic mechanisms, and the biochemical processes and major components underlying these mechanisms have been determined using advanced in vitro experiments. Two major types of RNA substitutions occur in mammals: A-to-I and C-to-U [18,19], whereas U-to-C substitution is found mainly in lower plants such as bryophytes (mosses and liverworts), lichens, fungi, and algae (including diatoms). Important biochemical differences between the processes involved in A-to-I and C-to-U substitutions have helped in understanding the mechanisms involved in C-to-U RNA editing and in the development of adjustments to control target specificity. A-to-I and C-to-U RNA editing are generally mediated by the ‘adenosine deaminase acting on RNA’ (ADAR) and ‘activation-induced cytidine deaminase/apolipoprotein B mRNA-editing enzyme catalytic polypeptide-like’ (APOBEC-AID) deaminase families, respectively; however, the enzymes responsible for U-to-C editing have yet to be discovered, although it is an abundant phenomenon in lower plant species.

C-to-U RNA editing is an important mechanism for regulated amplification of mammalian genetic diversity. The capacity to specify and restrict access to the RNA editing machinery is essential for this adaptation to be effective because it prevents the enzymatic modification of undesired targets. Identification of the core components of the apoB RNA editing holoenzyme, APOBEC, and examination of the functions of new candidate genes that encode other elements of the larger RNA editing complex will likely reveal further details regarding the processes involved in RNA metabolism.

In this study, we assessed artificial RNA editing systems using fibroblast cells derived from the mottled mutated macular mouse model [20], which is an animal model for Menkes disease where the T>C mutation is present in a particular gene. Menkes disease, also referred to as Menkes kinky hair disease, is a recessive X-chromosome-linked hereditary disease in humans caused by mutations (serine to proline) in a gene encoding the copper transport protein ATP7A, which lead to defective copper metabolism [21,22]. Characteristics of Menkes disease include developmental delay, peculiar hair structure, and neurological symptoms attributable to cerebral and cerebellar degeneration [23,24,25,26].

The gene causing Menkes disease was isolated by positional cloning [27,28,29]. Menkes protein, *ATP7A*, is a copper-transporting P-type ATPase that utilizes energy generated by ATP hydrolysis to move Cu(I) across cell membranes. All tissues except liver express the *ATP7A* transmembrane protein. By transferring Cu(I) from the small intestine into the blood, *ATP7A* controls Cu(I) absorption in the human body; hence, mutations of the *ATP7A* gene result in poor physiological dispersion of copper ions to cells [30], leading to copper aggregation in certain tissues, such as the small intestine and kidneys. The signs and symptoms of Menkes disease and occipital horn disease are caused by copper-containing enzymes displaying diminished activity [31].

The mottled mouse has been considered as an animal model for human Menkes kinky disease, an X-linked disorder of copper transport [32,33]. The macular mottled mutant mouse emerged in the C3Hf mouse strain and was expected to have a mutation in the *Atp7a* gene, as discovered in the dappled and the blotchy mottled mouse strains; however, northern and Southern analyses of the *Atp7a* gene revealed no gross variations between the wild-type and macular mouse strains [22,34,35]. The mutation of *Atp7a* in macular mice was first observed in cDNA prepared from spleen RNA and confirmed by investigation of genomic DNA using a polymorphic *Bam*HI site destroyed by the mutation [22]. Sanger sequencing analysis of cDNA demonstrated the presence of the T-to-C mutation in spleen and liver samples from 7-day-old hemizygous male mice.

Phenotypic features of the macular mouse contributing to its use as a model for Menkes disease include the development of white fur color and curly whiskers in hemizygote males from around postnatal day 3, along with reduced body weight from days 7 to 10 and death at approximately day 15, although the body weight of hemizygotes was almost the same as that of wild-type male littermates at 7 days old [36,37,38].

RNA–protein conjugate, the MS2 bacteriophage coat protein, and a stem-loop structure from the phage genome naturally interact to form MS2 tagging, which is used to biochemically purify RNA–protein complexes and conjugated with protein to detect RNA in living cells. This system is called the MS2 system (Supplementary Figure S4) In this MS2 system, the MS2 coat protein is fused to the deaminase domain of APOBEC, while the MS2 stem-loop RNA is fused to a 21 nt guide RNA, ultimately with the help of the coat protein stem-loop binding the deaminase, which can reach to the targeted sequence with the aid of guide RNA. With the help of the deaminase, this MS2 system is being used as a tethering agent over a longer period of time as a common editing approach. Previously in our laboratory, we applied a 6× MS2 system containing guide RNA for A-to-I editing with ADAR 1 deaminase [39,40,41,42] and C-to-U editing with the APOBEC 1 deaminase domain [43]. Further, a double MS2 system (1× MS2 stem-loop on either side of the guide RNA) has previously been applied along with ADAR 1 deaminase for A-to-I editing [44]. Following Katrekar and Mali (2019), [45] who described the double MS2 system, Bhakta et al. conducted experiments to restore A-to-I mutations in the genetic code using the MS2 system alongside ADAR 1 deaminase and guide RNA and found that the double MS2 system was more successful than the 6× stem-loop approach [44]. While co-transfection of two distinct factors (deaminase and guide RNA) is conducted on the cells, it may cause the cells to be more stressed, and it is also possible that the uniform release of both the factors will not occur (disbalance of ratio between the factors), which means the deaminase may be released but not the same amount or at the same time as the guide RNA, which will undoubtedly have an adverse impact on editing (reduced). As a result, we preferred to incorporate both the editing factors (APOBEC1-MS2 coat protein and guide RNA-MS2-stem-loop) into one single plasmid vector to make transfection easier and less stressful for the cells and also to ensure the proper release (uniformity) of the editing factors. There are several other techniques that are used for C-to-U editing, most of which are derivatives of CRISPR, such as CURE (dCas13X and APOBEC3), RESCUE, or RESCUE-S (dCas13X and mutated ADAR). But for CRISPR, we believe that the gene size requirement is large, which may be a disadvantage because of its immune response. This is a drawback for such systems when using vectal vectors to introduce genes in the future, and the immune response may limit its effectiveness. However, CRISPR can be conducted with nucleic acid strand breaks like Cas or with deaminases instead of Cas. But, with cleavage, the risk of off-target editing is higher.

In this study, we attempted to restore the genetic code or correct C-to-U mutation in macular-mouse-derived fibroblasts using APOBEC1 deaminase–MS2 coat protein with guide RNA and MS2 stem-loops, including the standard 6× MS2 stem-loops and a double MS2 system, with an MS2 stem-loop on either side of the guide RNA. In addition, we developed a single construct comprising both the editing factors (deaminase and guide RNA) in a single plasmid vector.

## 2. Materials and Methods

### 2.1. Ethical Approval

For the macular mouse experiment, ethical approval was received from the committee of animal experiments of JAIST, Japan, and it was Animal01-001 (01is Reiwa1, the year 2019).

To create the mutated mouse model for menkes kinky disease, macular mice were propagated by mating heterozygous females with wild-type males and maintained in an animal house with a constant temperature (22 °C) and a 12 h light/12 h dark cycle. Tap water and a commercial stock diet (Japan CLEA CE-2) were provided ad libitum. Hemizygous male mice were used as experimental animals, with wild-type male and heterozygous female littermates as controls. All the mice were weighed on days 7, 10, and 14 postpartum (Supplementary Figure S1).

### 2.2. Copper Therapy of Hemizygous Macular Mice

Hemizygous male mice were injected with copper ions to keep them alive (Supplementary Figure S1). Copper ions were administered to the mice by sub-cutaneous injection of copper chloride solution (10 mg of copper/g body weight in 0.9% NaCl) using a 2 mL Terumo micro syringe MT-2 (Tokyo, Japan) in the mid-dorsal region on the mornings of postnatal days 7 and 10.

### 2.3. Sample Collection

Mice were weighed and sacrificed by cervical dislocation on day 7 postpartum. The liver and spleen were removed immediately, weighed, and frozen at −80 °C until they were processed for further experiments. At the same time, primary fibroblast cells were immediately set in the culture after collecting them from the thigh regions.

### 2.4. RNA Extraction and cDNA Synthesis

Frozen liver and spleen samples were thoroughly ground using a gel grinder. Then, TRIzol (Invitrogen, Carlsbad, CA, USA) was added (500 μL/tube) along with chloroform (100 μL/tube), before the mixture was vortexed (5 min, full speed). Next, the samples were centrifuged (12,000× *g*, 15 min), supernatants collected into new tubes, and 500 μL isopropanol was added to each tube, followed by brief vortexing and a further centrifugation step (12,000× *g*, 10 min). RNA was then visible at the base of the tube, supernatants were removed, and 500 μL 70% ethanol was gently added to each tube, followed by a final centrifugation (8000× *g*, 5 min). Supernatants were removed and samples air dried. Then, RNA was dissolved in 20 μL TE buffer and the concentration was measured using a NanoDrop spectrometer (Thermo Scientific ND 1000, Waltham, MA, USA). RNA aliquots (500 ng) were used for cDNA synthesis with Superscript^TM^ III (Invitrogen), following the manufacturer’s protocol. Finally, cDNA concentration was measured using the ND-1000.

### 2.5. Identification of the Target Mutated C nt in Atp7a

The *Atp7a* gene was amplified from macular mouse model liver and spleen samples by PCR using the synthesized cDNA as a template and specific primers (forward, CTGGATGTTGTGGCAAGTATTGAC; and reverse, GCTGTTCAGGGAGCGCTTG) to generate a fragment of 466 bp (Supplementary Figure S2 and Table 1).

### 2.6. Confirmation of the Atp7a T-to-C Mutation by Sequencing

After PCR amplification of the *Atp7a* gene fragment, the reactions were separated by electrophoresis, the bands were excised from the gel, and the DNA was purified using a gel purification kit (QIAGEN gel purification kit, Hilden, Germany). The purified samples were sequenced using the Sanger sequencing method, and sequence data were analyzed using Applied Biosynthesis software 3130xl GeneticAnalyzer, Foster City, CA, USA to verify the exact location of the mutation (Supplementary Figure S2).

### 2.7. APOBEC 1 Deaminase Enzyme Preparation

**Figure 1:**
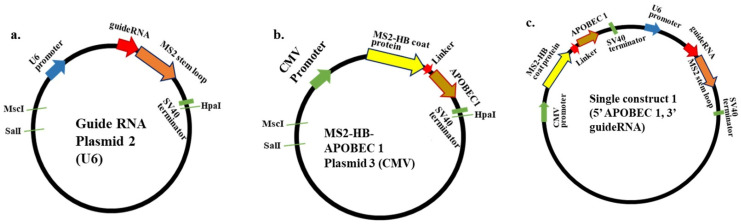
(**a**). Guide RNA Plasmid 2 (U6): Guide RNA construct under the control of the pol III U6 promoter. (**b**). MS2-HB-APOBEC 1 Plasmid 3 (CMV): APOBEC 1 deaminase construct under the control of pol II CMV promoter. (**c**). Single construct 1 (5′-APOBEC 1, 3′-guide RNA): Single construct, with the guide portion under the control of the pol III U6 promoter and the APOBEC 1 deaminase portion under the control of the pol II CMV promoter. Here, the CMV promoter is located upstream of the U6 promoter.

To enable direct enzyme targeting of the *Atp7a* target codon of interest, the deaminase domain of APOBEC1 was cloned downstream of MS2 in pCS2+MT, using XhoI and XbaI (Takara, Shiga, Japan), to yield pCS2+MT-MS2HB-APOBEC 1, following PCR amplification from the HEK 293 and HeLa cell lines using forward and reverse primers containing specific restriction sites, as follows: XhoI catalytic APOBEC1 forward, tccactcgagatgccctgggagtttgacgtctt; XbaI catalytic APOBEC1 reverse 1, acggtctagattaagggtgccgactcagaaactc; XbaI catalytic APOBEC1 reverse 2, acggtctagattattaagggtgccgactcagaaactc. Positive colonies were picked and confirmed by Sanger sequencing. The frame of the domain-encoding region sequence was confirmed using the ExPASY Bioinformatics resource portal and NCBI-BLAST searches (Figure 1).

### 2.8. Preparation of Guide RNA

The U6 promoter is predominantly used to express small nuclear RNAs and is suitable for the expression of guide RNAs, and its expression capacity is stronger than that of the CMV promoter. Further, Azad et al. noted that it is important to maximize guide RNA expression to increase editing efficiency [46]. Previously, Bhakta et al. used the pol II CMV promoter for C-to-U RNA editing [44]; however, transcription from pol II promoters requires a poly-A site for correct mRNA termination and processing, while pol III promoters (such as the U6 promoter) do not require polyadenylation and thus are ideal for generating small nuclear RNAs. Based on these data, we used a U6 promoter to drive guide RNA expression in this experiment. To prepare the guide RNA, a 21 nt sequence complementary to the target mRNA, but with a mismatched A at the target C position, was inserted upstream of MS2-RNA by adding the guide sequence using the PSL-MS2-6X (pCS2+guide RNA-MS2-6XStem-loop) forward primer and ligating with the pCS2+Only vector plasmid for expression under the control of the pol III U6 promoter (Figure 1). The following sequence was used:

atca**GAATTC***ATTGCTGCGGATCCCATCCAG*GAATGGCCATG, where the ‘atca’ tetrant leader sequence allows proper recognition by the restriction enzyme, the bold text indicates the restriction site, italic text represents the 21 nt guide sequence, and the underlined text indicates the forward primer for MS2-6X. The reverse MS2-6X primer was attc**CTCGAG**CGCAAATTTAAAGCGCTGAT (XhoI). Positive colonies were picked and confirmed by Sanger sequencing (Supplementary Figure S3, and Figure 1).

### 2.9. Preparation of a Single Construct 1 Encoding APOBEC 1 Deaminase and Guide RNA Under the Control of the Pol II CMV and Pol III U6 Promoters, Respectively

For the preparation of a single construct 1 (single construct 1), the entire APOBEC 1 deaminase coding sequence, along with that of the MS2-HB coat protein, under the control of the pol II CMV promoter and followed by an SV-40 terminator, were inserted into a vector construct containing guide RNA under the control of the pol III U6 promoter. The CMV-MS2HB-APOBEC1-SV40 terminator region was excised from the APOBEC1 construct using the NdeI and HpaI restriction enzymes. After digestion, both the vector and the insert ends were blunted using a Klenow fragment kit (Takara). The blunt-ended vector and insert were then ligated; the direction of the ligated fragments was not important, as each part had their own individual promoter and terminator sequences. The final construct was:

pCS2+CMV+MS2HB+APOBEC1+SV40+U6+guide RNA+MS2 6Xstem-loop+pCS2-Only.

After transformation into the *Escherichia coli* DH5α competent cells, plasmids were extracted from positive colonies using a QIAGEN Plasmid Midi Kit (QIAGEN, Germany), and the final construct was confirmed by sequencing, followed by BLAST searches (Figure 1).

### 2.10. Preparation of 1× MS2 Flanking Each Side of the Guide RNA (Double MS2 Guide RNA)

For the preparation of constructs with 1× MS2 on either side of the guide RNA, for fragments, oligos were designed following Katrekar et al., 2019 [45]. Oligos were then annealed and dephosphorylated using T4 polynucleotide kinase and 10X kinase buffer (New England Biolabs). Plasmid containing the pol III U6 promoter was digested with BbsI (New England Biolabs), and the digested plasmid was treated with BAP (bacterial alkaline phosphatase). Finally, three-way ligation of the two annealed products and the digested vector was conducted. Next, the ligated product was transformed into *Escherichia coli* DH5α competent cells, and the positive colonies were collected. Plasmid DNA was extracted using a QIAGEN Mini kit (QIAGEN) and then sequenced (Supplementary Figure S3, and Figure 1).

The guide RNA design (21 nt with an A mismatch at the target position and 1× MS2 on either side on the either side of the guide RNA) is shown below:

ACATGAGGATCACCCATGTCATTGCTGCGGATCCCATCCAGAACATGAGGATCACCCATGTC
Green, 1× MS2 stem-loop; sequence pink, complementary to the target; yellow, mismatched base.

### 2.11. Collection and Culture of Tail Fibroblasts

The Atp7a gene is expressed in various tissues, including fibroblasts; therefore, tail fibroblasts were collected from mice. Hemizygous male macular mice (7 days old) were sacrificed by cervical dislocation. Then, the tails of the mice were immediately severed and washed in a Petri dish containing ice-cold phosphate buffered saline (PBS). Next, pieces of tail were placed in a 35 mm dish containing DMEM supplemented with 1% streptomycin and penicillin (SP) and 10% fetal bovine serum (FBS) and gently chopped using scissors, to allow the cells to easily attach to the bottom of the dish. After 3 days, the media was swapped again for fresh DMEM with 1% SP and 10% FBS. After 7 days, when the cells had grown and formed a network, they were sub-cultured and prepared for transfection. Cells were seeded for electroporation after the second or third passage.

### 2.12. Electroporation of Cells for Genome Editing

Approximately 0.5 million fibroblasts were cultured in a 60 mm dish (Sarstedt AG & Co. KG, Nümbrecht, Germany #83.3911.002), trypsinized, pelleted by gentle centrifugation (Thermo Scientific #75004250), and resuspended in 900 μL electroporation buffer. Aliquots (100 µL; equivalent to 0.3 × 10^6^ cells) were mixed with plasmid DNA (conc. 800 ng/uL) and transferred into electroporation cuvettes (2 mm; Cell Projects, #EP-102) for pulsing; various parameters (voltage, pulse duration, plus number, and interval between pulses) were used for square wave pulses. Pulses were recorded online, and a drop of 2–3% of the set voltage was typically measured. For every experiment, a handling control (100 μL of cell suspension seeded in one of the wells of a 6-well plate) and a mock control (100 μL of cell suspension electroporated without plasmid) were performed. After electroporation, cells were transferred into one well of a six-well plate (Sarstedt AG & Co. KG, #83.3920.300), the culture medium was added, and the plate was placed in an incubator. Electroporation medium was swapped for standard culture medium after 24 h, and cell viability and fluorescence were assessed. Various conditions were tested to assess their effects on transfection efficiency by electroporation, including the electroporation buffer, DNA concentration, and electroporation pulse parameters such as voltage, number and duration of pulse, and cuvette type and temperature. The electroporation buffers used were Gene Pulser Electroporation Buffer (Biorad, CA, USA, #165–2677), Opti-MEM Reduced Serum Medium with GlutaMax Supplement (ThermoFisher, MA, USA #51985–026), and Dulbecco’s PBS (Sigma-Aldrich, MA, USA, D5652). All electroporation steps were conducted at room temperature (Supplementary Figure S6). Not only the editing factors but also the pcDNA-3EGFP plasmid construct was electroporated into the mice-derived fibroblast cells. The electroporation conditions were as follows: **Poring Pulse****Transfer pulse****Voltage**120 V15 V**Pulse length**0.5 ms50 ms**Pulse Interval**50 ms50 ms**Pulse Number**22

### 2.13. RNA Extraction from Electroporated Cells and cDNA Synthesis

After 48 h of electroporation, transfected cells grown in dishes or plates were first rinsed with ice-cold PBS. Then, RNA was extracted using TRIzol^(R)^ reagent (Invitrogen, Carlsbad, CA, USA), followed by cDNA synthesis with the Superscript^TM^ III First stand synthesis system (Invitrogen), according to the manufacturer’s protocol.

### 2.14. PCR Amplification and Sanger Sequencing

The primers, CTGGATGTTGTGGCAAGTATTGAC (forward) and GCTGTTCAGGGAGCGCTTG (reverse), were used for the PCR of Atp7a, with synthesized cDNA as a template. The total length of the amplified fragment was 466 bp, Table 1.

### 2.15. Confirmation of Sequence Editing

After PCR amplification, amplicons were separated in 6% polyacrylamide gel by loading equal volumes (3 μL) of PCR product into each well. Gels were observed using an LAS 3000 gel imager, Fujifilm, Tokyo, Japan, the bands were excised, and the DNA was purified using a Qiagen Gel Extraction kit (Hilden, Germany). Then, purified samples were sent for Sanger sequencing. Sequence data were analyzed and editing rates calculated based on peak height and peak area, as follows:

$$\begin{array}{l} Editing\;efficiency\;\left(sense\right)=\\ \;Considering\;peak\;area:\;\frac{\mathrm{Area}\;\mathrm{of}\;T}{\mathrm{Area}\;\mathrm{of}\;T\;+\;\mathrm{Area}\;\mathrm{of}\;C}\times 100\\ Considering\;peak\;height:\frac{\mathrm{Peak}\;\mathrm{height}\;\mathrm{of}\;T}{\mathrm{Peak}\;\mathrm{height}\;\mathrm{of}\;T\;+\;\mathrm{Peak}\;\mathrm{height}\;\mathrm{of}\;C}\times 100 \end{array}$$

### 2.16. Determination of Cytochrome c Oxidase Activity

Cytochrome c oxidase activity in mouse-derived fibroblasts was detected using a Cytochrome Oxidase Activity Assay Kit (Colorimetric) (Abnova); for transfected fibroblasts, cytochrome c oxidase activity alteration was considered to validate the transfection. First, mitochondrial proteins were extracted from fibroblasts using a Mitochondrial/Cytosol fraction kit (Abnova, Taipei City, Taiwan). Then, cytochrome c oxidase activity was assayed in the protein samples using a spectrophotometer (SmartSpec^TM^ Plus Spectrophotometer, BioRAD, CA, USA) set at 550 nm with kinetic function (pulse duration, 30–240 s; interval, 30 s). Optical density (OD) values were read at each time point. ΔOD and ΔT were calculated, followed by cytochrome c oxidase activity, as follows:

$$Cytochrome\;c\;oxidase\;\left(units/mg\right)=\frac{\Delta OD/\Delta T}{\in \times protein\;\left(\mathrm{mg}\right)}$$

where ε is 7.04 mM^−1^cm^−1^ (molar extinction coefficient of reduced cytochrome c at 550 nm) and units are nmol/min/mg protein.

### 2.17. Statistical Analysis

Data are presented as mean ± standard error (SE). The SE was computed from known sample statistics, providing an unbiased estimate of the standard deviation of the statistic. Differences in copper concentration, CCO activity, and catecholamine between the two groups were analyzed using one-way repeated measure ANOVA (Tukey post hoc test); *p* < 0.05 was considered significant.

## 3. Results

### 3.1. Confirmation of the Atp7a Mutation in Macular Mice and Restoration of the Genetic Code

The MS2 system, along with the APOBEC 1 deaminase and guide RNA plasmids, were transfected into fibroblasts derived from macular mice to examine the ability of this system to restore the *Atp7a* genetic code by artificial RNA editing. Three different types of guide RNAs were applied for genetic code restoration: a guide RNA with a U6 promoter with 6× MS2 stem-loops, a single construct 1 (5′ APOBEC1 and 3′ guide RNA) encoding both editing factors, and a double MS2 system (1× MS2 stem-loop flanking either side of the guide RNA).

No fluorescence-expressing genes were present in the transfected mouse-derived fibroblasts, nor was any fluorescence-encoding plasmid among those transfected for gene editing. Therefore, to verify the transfection efficiency, a plasmid encoding GFP was transfected in parallel, and fluorescence intensity was determined as a measure of transfection efficiency (Supplementary Figure S5).

There was no restriction site at the restored codon position; therefore, sequence restoration was solely determined by Sanger sequencing. Sanger sequencing analysis provided evidence of genetic code restoration. In mutated samples, only a single C peak was observed, but after transfection with APOBEC1 deaminase and guide RNA, editing occurred, and a dual peak, comprising the mutated C and restored T, was observed, demonstrating that the deaminase system successfully restored the genetic code at the T-to-C mutation site. When only one factor was transfected (i.e., either the APOBEC 1 deaminase or the guide RNA), only the mutated C peak was observed, and no restored T peak was visible (Figure 1).

**Figure 2:**
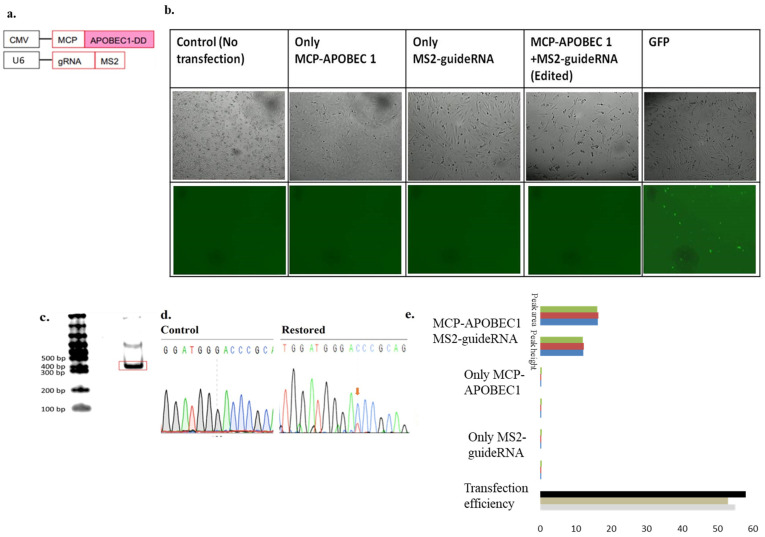
(**a**). Transfection of the CMV-promoted deaminase and U6-promoted guide RNA plasmids into the mice derived fibroblast cells, confirmed the restoration of genetic code. (**b**). Panel of transfection into the mouse-derived fibroblast cells, Images were taken at 200 um scale bar. (**c**). PCR amplification of the ATP7A gene from the synthesized cDNA of transfected cells. (**d**). Sanger’s sequencing showing the control and restoration of the genetic code. Restored peak is shown by the Orange arrow. (**e**). Calculation of the editing efficiency from the peak area and peak height and graph showing the restored percentage (editing percentage) from the peak area and peak height, respectively. The different colors express the different replicates. Statistical analysis (mean ± SEM) was conducted for all data (n = 3).

Editing efficiency was calculated based on peak area and peak height, determined from Sanger sequencing data using ImageJ software (NIH, MD, USA) and using EditR software, MN, USA. The results demonstrated that use of the APOBEC 1 deaminase and U6-21bp upstream-MS2-6X restored 12.1% and 16.25% of the genetic code, according to peak area and peak height, respectively (Figure 2 and Supplementary Figure S7).

### 3.2. Single Construct 1 Transfected into Mouse-Derived Fibroblasts

Next, a single construct 1 encoding both editing factors was transfected into fibroblasts derived from the macular mouse model by electroporation; the construct contained the U6 promoter to drive guide RNA expression and the CMV promoter for APOBEC1 expression, with the guide RNA being upstream of the MS2 stem-loop and the CMV promoter upstream of the U6 promoter.

**Figure 3:**
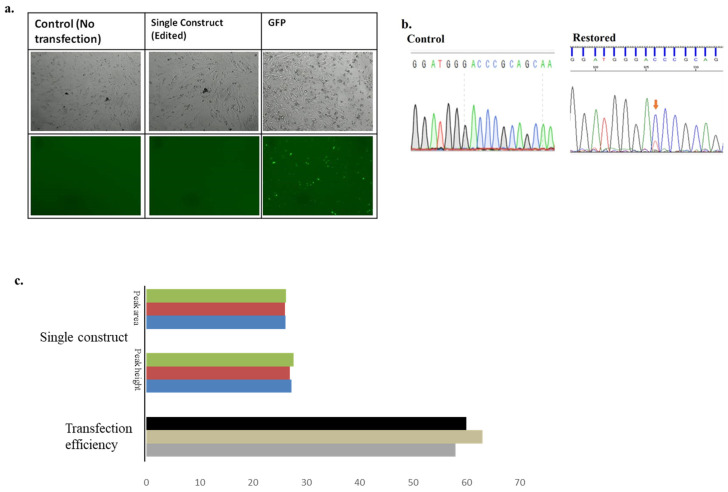
Confirmations of the restoration of the genetic code by using single construct 1. (**a**). Panel of transfection in mouse-derived fibroblast cells, Images were taken at 200 um scale bar. (**b**). Sanger’s sequencing showing the control and restoration of the genetic code. Restored peak is shown by the Orange arrow. (**c**). Calculation of the editing efficiency from the peak area and peak height, and graph showing the editing efficiency from the peak area and peak height, respectively. The different colors express the different replicates. Statistical analysis (mean ± SEM) was conducted for all data, where n = 3.

After transfection, RNA was extracted from the cells, followed by cDNA synthesis and PCR amplification. The amplified fragment was then subjected to Sanger sequencing analysis using a sense primer. The resulting sequence data again provided evidence of genetic code restoration using the single plasmid system. In mutated samples, only a single C peak was observed, but after transfection with the single construct 1 containing the APOBEC1 deaminase and guide RNA, editing occurred, resulting in a dual peak of mutated C and restored T, demonstrating that the deaminase system successfully restored the genetic code. Calculation of Sanger sequencing peak area and peak height demonstrated that the APOBEC 1 deaminase and U6-MS2-6X 21 upstream system restored 27.20% and 26.09% of the genetic code, respectively (Figure 3 and Supplementary Figure S7).

### 3.3. Application of Guide RNA with 6× MS2 Flanking Either Side of the Guide RNA (Double MS2-Guide RNA)

**Figure 4:**
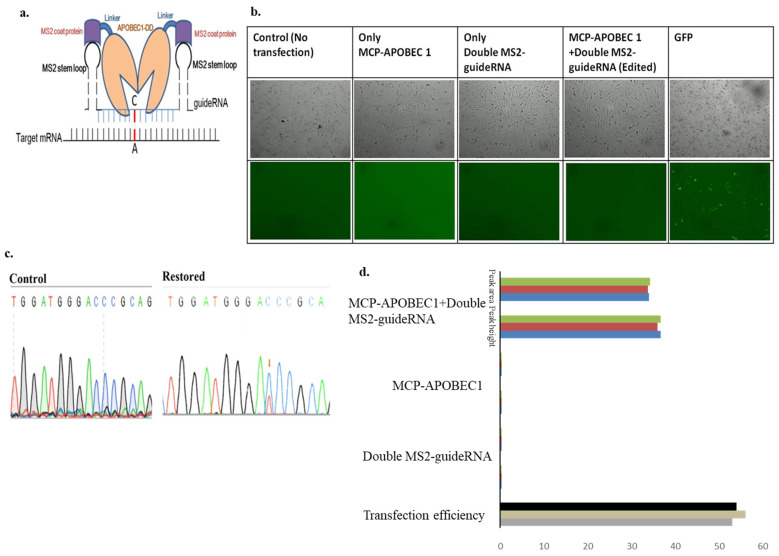
(**a**). Schematic model of the double MS2 stem-loop on either side of the guide RNA system for mediated editing by APOBEC1 deaminase. (**b**). Panel of transfection in mouse-derived fibroblast cells, Images were taken at 200 um scale bar. (**c**). Sanger’s sequencing confirmation of the control and C-to-U editing, achieved using the double MS2-guide RNA system along with MCP-APOBEC 1 deaminase in fibroblast cells derived from macular mouse tails, Restored peak is shown by the Orange arrow. (**a**). Sequence data showing restored T peak at the position of the mutated C peak. (**d**). Calculation of the editing rate from the peak area and peak height, respectively. The different colors express the different replicates. Statistical analysis (mean ± SEM) was conducted for all data, where n = 3.

The double MS2-guide RNA system basically includes the flanking part of the 6XMS2 stem-loop on either side of the guide RNA being promoted by the U6 promoter (Figure 4a).

Several different pulse conditions were used to optimize the conditions for the transfection of editing factors via electroporation. Sequence data again demonstrated the restoration of the T peak at the mutated C position. The editing rates of this system, calculated based on peak area and peak height, were 36.66% and 34%, respectively (Figure 4 and Supplementary Figure S7).

### 3.4. Detection of Cytochrome c Oxidase Activity

**Figure 5:**
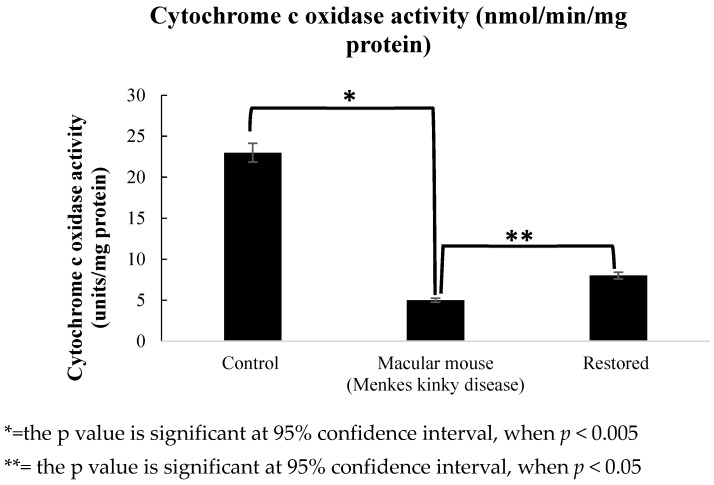
The graph shows the cytochrome c oxidase activity in control (fibroblast cells derived from normal litter mate), hemizygous macular mouse (fibroblast cells derived from hemizygous mouse), and the restored cells in which the editing factors (MCP-APOBEC1 and double MS2-guide RNA) were transfected. The cytochrome c oxidase activity significantly improved in the restored cells. Statistical analysis (One-Way ANOVA) was conducted for all data, where n = 3.

Cytochrome c oxidase is a copper-dependent enzyme whose activity is reduced in the cells of macular mice due to mutation in *Atp7a* resulting in physiological imbalance of copper metabolism. Hence, when the *Atp7a* mutation is restored by the application of the editing factors (deaminase and guide RNA), cytochrome c oxidase activity is expected to be improved. To validate our results, we performed a cytochrome c oxidase assay using control, macular mouse, and restored mouse fibroblast samples (in this case, MCP-APOBEC 1 and double MS2-guide RNA were transfected together, as the highest editing efficiency was achieved from this treatment). In fibroblasts from wild-type male littermates, cytochrome c oxidase activity was 23 units/mg protein, which was significantly higher than that of cells from hemizygous macular male mice, at 5 units/mg protein (*p* = 0.003). After the introduction of editing factors into mouse fibroblasts with mutated *Atp7a*, a proportion of the mutated C nt was altered into the non-mutated T base, resulting in restoration of ATP7A activity and consequent improvement of copper transport. Our data showed that some increase in cytochrome c oxidase activity was observed. The cytochrome c oxidase activity was significantly higher in cells subjected to RNA editing than that in untreated macular mouse fibroblasts, at 8 units/mg protein (*p* = 0.0213) (Figure 5).

## 4. Discussion

Sanger sequencing analysis provided evidence of genetic code restoration in the Atp7a gene. The presence of dual mutated C and restored T peaks in experimental samples demonstrated the successful restoration of the T-to-C mutation by the deaminase system. No restoration was detected when only one factor (either APOBEC1 deaminase or guide RNA) was transfected. Editing efficiency was calculated using both ImageJ (NIH) and EditR software, based on Sanger sequencing peak area and peak height. The combination of APOBEC1 deaminase and U6-21bp upstream-MS2-6× restored 12.17% and 16.25% of the genetic code, based on peak area and peak height, respectively. Transfection of single construct 1, containing both APOBEC1 deaminase and U6-MS2-6× 21 upstream, showed higher editing efficiencies of 27.20% and 26.09%, respectively.

The effectiveness found here for single construct 1, which contained both CMV and U6 promoters, aligns with findings by Su et al. (2008) [47]. They reported that a hybrid construct with the CMV promoter or enhancer placed immediately upstream of the U6 promoter increased shRNA silencing efficiency. This improved performance can be attributed to several factors: proper maintenance of the ratio between guide RNA and APOBEC1 deaminase, which may not occur when using individual constructs; reduced cellular stress compared to transfection with two separate factors; a simplified transfection process, as both components are present in the same plasmid construct; and enhanced editing efficiency due to the combination of pol III U6 and pol II CMV promoters, surpassing the performance of either promoter alone. These findings highlight the advantages of using a single construct approach for genetic code restoration in the Atp7a gene.

Subsequently, we introduced the 1× MS2 guide RNA (double MS2-guide RNA) along with the MCP-APOEBC1 deaminase and determined the editing rate, which was 36.66% and 34%, according to peak area and peak height, respectively. These results are supported by findings reported by Katrekar et al. (2019) [45] and Bhakta et al. (2021) [44], who found that the double MS2 system was superior to other stem-loop systems and that the addition of a nuclear export signal within the construct increased the editing rate further still [44,45]; however, previous studies were conducted for A-to-I editing, whereas this study was for C-to-U editing.

Cytochrome c oxidase activity was significantly higher in cells from hemizygous male macular mice after genetic code restoration compared to untreated cells, though still lower than in wild-type control male mice. These findings demonstrate that the Atp7a gene mutation leads to impaired copper metabolism, resulting in significantly reduced cytochrome c oxidase activity compared to control mice (*p* = 0.003). The application of the artificial deaminase system significantly increased cytochrome c oxidase activity in cells from hemizygous male macular mice (*p* = 0.023), attributable to the restoration of the genetic code. Our results align with previous studies, albeit with some differences: Rossi et al. (2001) [48] observed a slight decrease in cytochrome c oxidase activity in an animal model, using brain and liver cells rather than tail fibroblasts. Kogot-Levin et al. (2016) [49] found cytochrome c oxidase activity reduced to one third of control values in patient muscle, supporting the hypothesis that copper accumulates in a biologically non-active form in Menkes disease. However, they noted normal activity levels in cultured muscle cells and fibroblasts, possibly due to higher copper availability in culture media. Several factors may have influenced our cytochrome c oxidase results: The fibroblasts were isolated from 7-day-old hemizygous male mice that received copper ion injections for survival. The culture serum likely contained copper ions, potentially activating cytochrome c oxidase to some extent. However, these factors do not account for the genetic code restoration observed through Sanger sequencing. The high level of restoration in the mRNA sequence suggests increased synthesis of normal (wild-type) protein. While our results are promising, further research is needed, such as long-term animal studies to assess the treatment’s effectiveness in improving the condition and investigation of potential side effects or unintended consequences of the genetic restoration approach.

This investigation presents a critical step toward the improvement of the specificity and safety of RNA editing. A significant consideration for gene therapy by RNA targeting, especially using non-integrating vectors, is the need for intermittent re-administration of the effector constructs and other transcripts, owing to the typically limited half-lives of edited mRNA and effector molecules. Furthermore, relative to CRISPR Cas9-based editing approaches, the RNA-guided deaminase strategy is directly relevant to human therapeutics, because some versions solely utilize human effector RNAs and proteins. Additionally, as deaminases are widely expressed (for example, ADAR1 is endogenously expressed in most human tissues, ADAR2 is expressed in the lung and brain, and APOBEC1 is only found in the small intestine), human-endogenous recruitment of these molecules via guide RNAs bearing long-antisense domains presents a very attractive strategy for effective RNA editing.

For such approaches of artificial RNA editing, off-target effects might be a concern regarding the efficacy of the system. Our group has previously worked on the MCP-APOBEC 1 (MS2-coat protein-APOBEC 1 deaminase) system for BFP-to-GFP genetic code restoration in HEK 293 cells, where we conducted the NGS experiment to figure out the off-target effects in that case, and we found that off-target effects are not a major problem in the MCP-APOBEC1 and MS2-guide RNA system. The off-target effects are not very significant [43]. I do agree that, in the current study, the target has been changed, but the approach and the developed system are the same, so we assume that the outcome regarding the off-target effects will remain the same.

In X-linked recessively inherited diseases, carriers (female) usually do not develop the disease, but next-generation daughters can be either unaffected or carriers, and sons will become either affected or unaffected. As such, female carriers usually do not develop the disease but rather pass it on to the next generation (sons), who express the trait. In X-linked inherited diseases, the X chromosome is usually assumed to be randomly inactivated on one of the X chromosomes in females. But, if the ATP7A genes were randomly inactivated, about half of the carriers would develop the disease, and many more female patients would be found, but in fact, only few carriers develop the disease. In autosomal recessive forms of the disease, the absence (deletion) of one of the two copies of the gene will not cause the disease. Thus, it might be enough if half of the gene is expressed [50,51,52]. If this is the case, it can be assumed that if 50% of the mRNA was restored, patients with the disease might recover. Using our system, we were able to restore approximately 35% of the RNA in mouse-derived fibroblasts. Therefore, we expect that this newly developed system can be adopted as a means of potential therapy for such X-linked recessive diseases.

## 5. Conclusions

Following our findings, we anticipate that, with progressive improvement, this approach will have broad implications for diverse basic science and therapeutic applications. Several other effector systems, such as the CRISPR-Cas system, are of prokaryote origin, raising significant risk of immunogenicity for in vivo therapeutic applications. To avoid these limitations of DNA nucleases, approaches that instead directly target RNA would be highly desirable, as these would enable maintenance and reversibility, and most importantly, would not induce permanent off-target mutations. Additionally, unlike DNA, RNA can be targeted via simple RNA–nucleic acid hybridization. Thus, RNA base editing via human RNA-guided cytidine deaminases could be an attractive approach for in vivo correction of disease-causing point mutations.

## Figures and Tables

**Table 1:** The PCR primer sets used in the experiments.

Primer for	Forward	Reverse
Amplification of Atp7a gene from macular mouse tissue	CTGGATGTTGTGGCAAGTATTGAC	GCTGTTCAGGGAGCGCTTG
Preparing pCS2+MT-MS2HB-APOBEC 1		
XhoI catalytic APOBEC1	tccactcgagatgccctgggagtttgacgtctt	
XbaI catalytic APOBEC1 Reverse 1		acggtctagattaagggtgccgactcagaaactc
XbaI catalytic APOBEC1 Reverse 2		acggtctagattattaagggtgccgactcagaaactc

## Data Availability

The data generated or analyzed during this study can be found within the published article and its Supplementary Files.

## Supplementary Materials

The following supporting information can be downloaded at: https://www.mdpi.com/article/10.3390/biom15010136/s1, Figure S1: (a). Macular mouse reared in the Laboratory, (b). Body weight change along with days according to the genotype. The graph shows the body weight of the heterozygous female (Ml/+) and normal littermate male (+/y) along with the progress of the days; day 7, 10 and 14. For all data the statistical analysis (mean ± SEM) was done, where n = 3; Figure S2: (a). PCR amplification of the targeted portion from cDNA of Liver and Spleen hemizygous macular mouse, (b). Sequence confirmation of the T-to-C mutation in ATP7A gene from the collected Liver and Spleen samples; Figure S3: (a). Guide RNA sequence, 21 ntds of guide RNA and 6X-MS2 stem loop, (b). Sequence result of the prepared Double MS2 guide RNA (1X MS2 stem loop on the either side of the guide RNA sequence); Figure S4: Schematic model of the MS2-APOBEC1 system where the MS2-stem loop is attached with the guide RNA and the MS2-Coat Protein is fused with the APOBEC 1 deaminase, transfection of the system into the mice fibroblast cells do the editing; Figure S5: (a). Fluorescence image showing the transfection efficiency while the wild type GFP was transfected into the macular mouse tail derived fibroblast cells having ATP7A gene, (a’). On the contrary when the APOBEC 1 and guide RNA was transfected into the macular mouse tail derived fibroblast cells having mutated ATP7A gene there was no fluorescence expression as there was no expressing gene, image was taken by Juli fluorescence microscope, (b). Transfection efficiency of the electroporation was calculated from the fluorescence intensity after application of the only wild type GFP into the macular mouse tail derived fibroblast cells without other editing factors, n = 3, (c). PCR amplification of the targeted ATP7A gene from the predicted restored sample, transfected with the editing deaminase APOBEC 1 and guide RNA; Figure S6: Collection of the Fibroblast cells from Macular mouse and condition of electroporation along with the editase and guideRNA; Figure S7: Panel of sequencing result, a.: control (only fibroblast cells no transfection), b.: MCP-APOBEC1 Only, c.: MS2-guide RNA Only, d.: MCP-APOBEC1+MS2-guideRNA (Edited), e.: Single construct, and f.: MCP-APOBEC1+Double MS2 (guide RNA).
