# Supplementary material for: Restoration of Genetic Code in Macular Mouse Fibroblasts via APOBEC1-Mediated RNA Editing
Source: Biomolecules. 2025 Jan 16;15(1):136. doi: 10.3390/biom15010136 (PMC11762822; doi:10.3390/biom15010136)
Supplement: Supplementary file 1 [file biomolecules-15-00136-s001.zip › biomolecules-3364191-supplementary.pdf]

Supplementary Data Set

Restoration of Genetic Code in Macular Mouse Fibroblasts via APOBEC1-Mediated RNA Editing

Supplementary Figure S1

a.

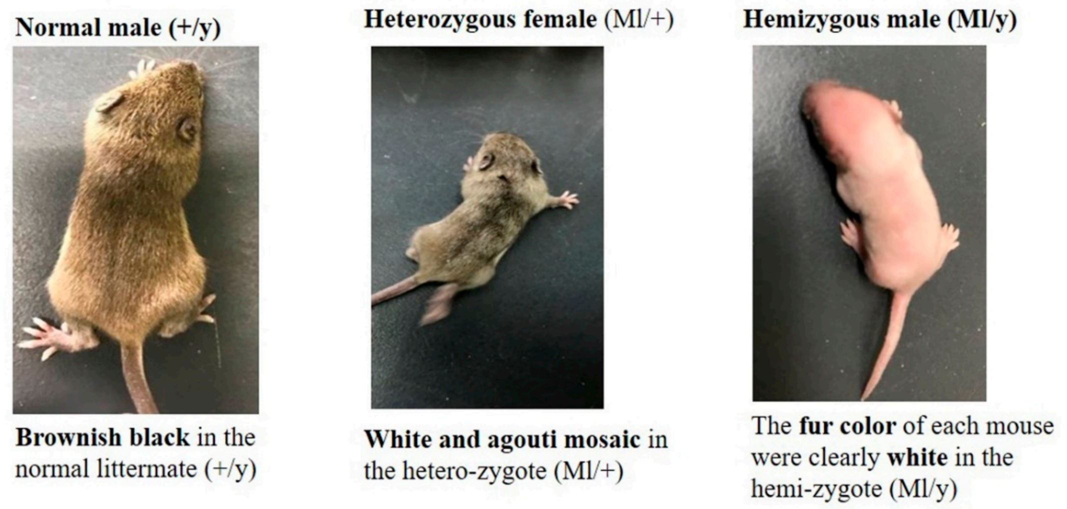

b.

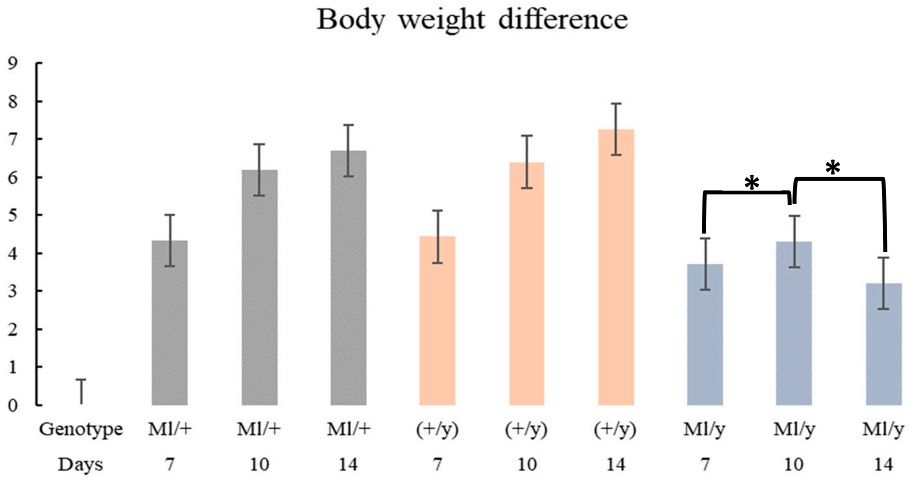

*=the p value is significant at 95% confidence interval, as p< 0.05

**= the p value is significant at 95% confidence interval as p<0.01

Supplementary Figure S1:

a. Macular mouse reared in the Laboratory
b. Body weight change along with days according to the genotype. The graph shows the body weight of the heterozygous female (Ml/+) and normal littermate male (+/y) along with the progress of the days; day 7, 10 and 14. For all data the statistical analysis (mean±SEM) was done, where n=3.

Supplementary Figure S2

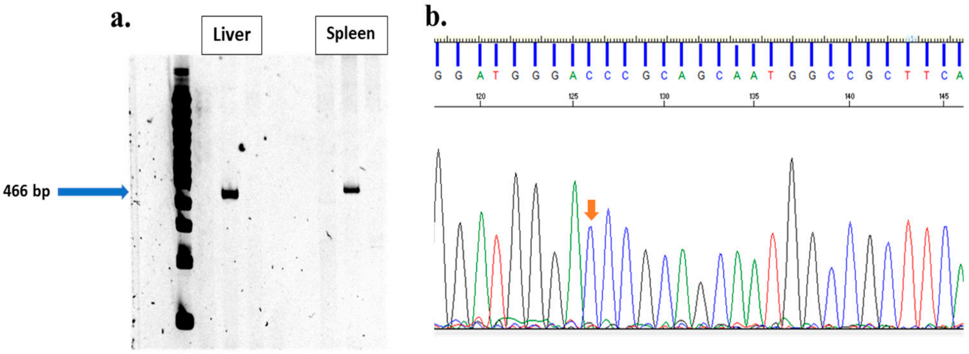

Liver:
GCTCTAATTTATAATCTGGTTGGAATTCCCATCGCTGCTGGAGTTTTCCTGCCCATCGGTTTGGTTTTACAACCCTGGATGGGA**C**CC
GCAGCAATGGCCGCTTCATCTGTCTCTGTAGTCCTTTCTTCCCTTTTCCTCAAGCTTTACAGGAAACCTACGTATGACAATTATGAG
TTGCATCCCCGGAGCCACACAGGACAGAGGAGTCCTTCAGAAATCAGTGTTCACGTTGGAATAGATGACACCTCAAGAAATTCTCC
AAGGCTGGGTTTGCTGGACCGGATTGTCAACTATAGCAGAGCCTCCATAAATTCACTGCTGTCTGACAAGCGCTCCCTGAACAGC

Spleen:
AATAATATATAATCTGGTTGGAATTCCCATCGCTGCTGGAGTTATCCTGCCCATCGGTTTGGTTTTACAACCCTGGATGGGA**C**CCGC
AGCAATGGCCGCTTCATCAGTCTCTGTAGTCCTTTCTTCCCTTTTCCTCAAGCTTTACAGGAAACCTACGTATGACAATTATGAGTT
GCATCCCCGGAGCCACACAGGACAGAGGAGTCCTTCAGAAATCAGTGTTCACGTTGGAATAGATGACACCTCAAGAAATTCTCCAA
GGCTGGGTTTGCTGGACCGGATTGTCAAC

Supplementary Figure S2: **a**. PCR amplification of the targeted portion from cDNA of Liver and Spleen hemizygous macular mouse
b. Sequence confirmation of the T-to-C mutation in ATP7A gene from the collected Liver and Spleen samples

Supplementary Figure S3

a.

GAATTC**ATTGCTGCGGATCCCATCCAG**GAATGGCCATGGGACGTCGACC
TGAGGTAATTATAACCCGGGCCCTATATATGGATCCTAAGGTACCTAATTGC
CTAGAAAACATGAGGATCACCCATGTCTGCAGGTCGACTCTAGAAAACAT
GAGGATCACCCATGTCTGCAGTATTCCCGGGTTCATTAGATCCTAAGGTACC
TAATTGCCTAGAAAACATGAGGATCACCCATGTCTGCAGGTCGACTCTAGA
AAACATGAGGATCACCCATGTCTGCAGTATTCCCGGGTTCATTAGATCCTAA
GGTACCTAATTGCCTAGAAAACATGAGGATCACCCATGTCTGCAGGTCGAC
TCCAGAAAACATGAGGATCACCCATGTCTGCAGTATTCCCGGGTTCATTAG
ATCTGCGCGCGATCGATATCAGCGCTTTAAATTTGCGCTCGAG

b.

GGCTGTTAGAGAGATAATTGGAATTAATTTGACTGTAAACACAAAGATATTAGTACAAAATACGTGACGTAGAAAGTAATAATT
TCTTGGGTAGTTTGCAGTTTTAAAATTATGTTTTAAAATGGACTATCATATGCTTACCGTAACTTGAAAGTATTTCGATTTCTTG
GCTTTATATATCTTGTGGAAAGGACGAAACACCGAACATGAGGATCACCCATGTCATTGCTGCGGATCCCATCCAGAACATGA
GGATCACCCATGTCTTTTTTATGCATGTAATACGGTTATCCACAGAATCAGGGGATAACGCAGGAAAGAACATGTGAGCAAAA
GGCCAGCAAAAGGCCAGGAACCGTAAAAAGGCCGCGTTGCTGGCGTTTTTCCATAGGCTCCGCCCCCCTGACGAGCATCAC
AAAAATCGACGCTCAAGTCAGAGGTGGCGAAACCCGACAGGACTATAAAGATACCAGGCGTTTCCCCCTGGAAGCTCCCTC
GTGCGCTCTCCTGTTCCGACCCTGCCGCTTACCGGATACCTGTCCGCCTTTCTCCCTTCGGGAAGCGTGGCGCTTTCTCATA
GCTCACGCTGTAGGTATCTCAGTTCGGTGTAGGTCGTTCGCTCCAAGCTGGGCTGTGTGCACGAACCCCCCGTTCAGCCCGA
CCGCTGCGCCTTATCCGGTAACTATCGTCTTGAGTCCAACCCGGTAAGACACGACTTATCGCCACTGGCAGCAGCCACTGGT
AACAGGATTAGCAGAGCGAGGTATGTAGGCGGTGCTACAGAGTTCTTGAAGTGGTGGCCTAACTACGGCTACACTAGAAGAA
CAGTATTTGGTATCTGCGCTCTGCTGAAGCCAGTTACCTTCGGAAAAAGAGTTGGTAGCTCTTGATCCGGCAACA

Supplementary Figure S3:

a. Guide RNA sequence, 21 ntds of guide RNA and 6X-MS2 stem loop Green Highlighted parts are the two restriction sites EcoRI and XhoI, **Bold** and Blue Highlighted part is the guide RNA and the ash color Highlighted part is the MS2-6X stem loop
b. Sequence result of the prepared Double MS2 guide RNA (1X MS2 stem loop on the either side of the guide RNA sequence)

Here yellow Highlighted part is the U6 promoter, Green Highlighted parts are the two 1X MS2 stem loop on the either side of the guide RNA, pink highlighted part is the guide RNA, rest is the backbone of pCS2+Only plasmid vector

Supplementary Figure S4

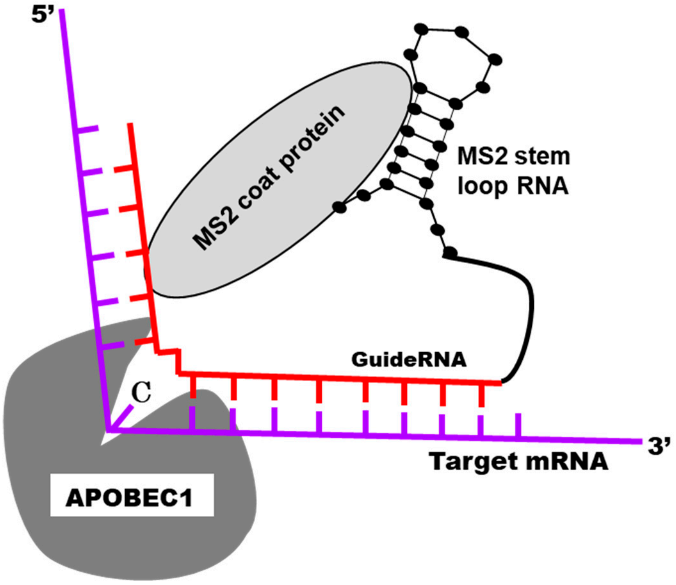

Supplementary Figure S4:

Schematic model of the MS2-APOBEC1 system where the MS2-stem loop is attached with the guide RNA and the MS2-Coat Protein is fused with the APOBEC 1 deaminase, transfection of the system into the mice fibroblast cells do the editing.

Supplementary Figure S5

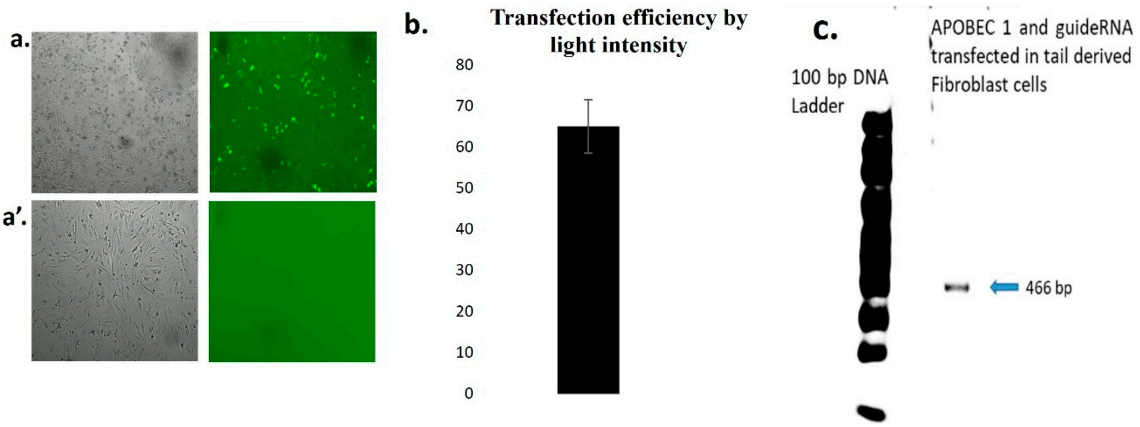

Supplementary Figure S5:

a. Fluorescence image showing the transfection efficiency while the wild type GFP was transfected into the macular mouse tail derived fibroblast cells having ATP7A gene,

a'. On the contrary when the APOBEC 1 and guide RNA was transfected into the macular mouse tail derived fibroblast cells having mutated ATP7A gene there was no fluorescence expression as there was no expressing gene, image was taken by Juli fluorescence microscope

b. Transfection efficiency of the electroporation was calculated from the fluorescence intensity after application of the only wild type GFP into the macular mouse tail derived fibroblast cells without other editing factors, n=3

c. PCR amplification of the targeted ATP7A gene from the predicted restored sample, transfected with the editing deaminase APOBEC 1 and guide RNA.

Supplementary Figure S6

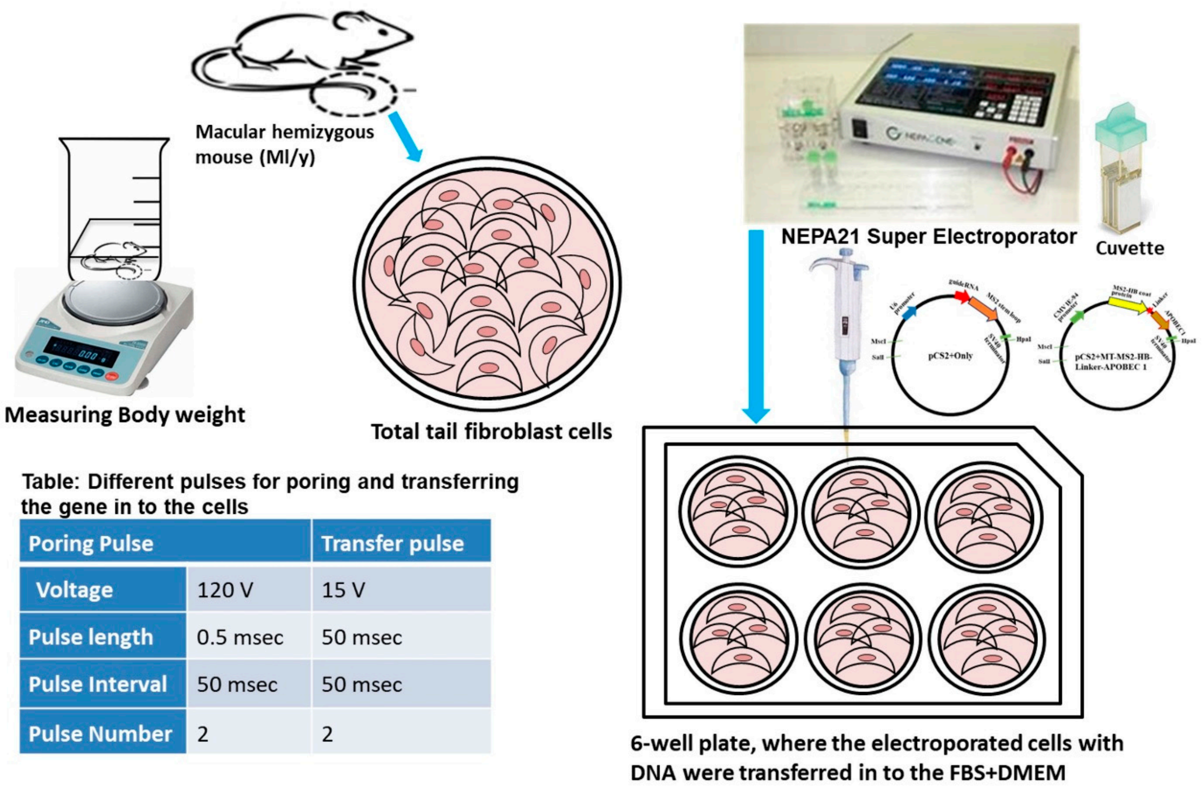

Poring Pulse		Transfer pulse
Voltage	120 V	15 V
Pulse length	0.5 msec	50 msec
Pulse Interval	50 msec	50 msec
Pulse Number	2	2

Supplementary Figure S6:

Collection of the Fibroblast cells from Macular mouse and condition of electroporation along with the editase and guideRNA.

Supplementary Figure S7

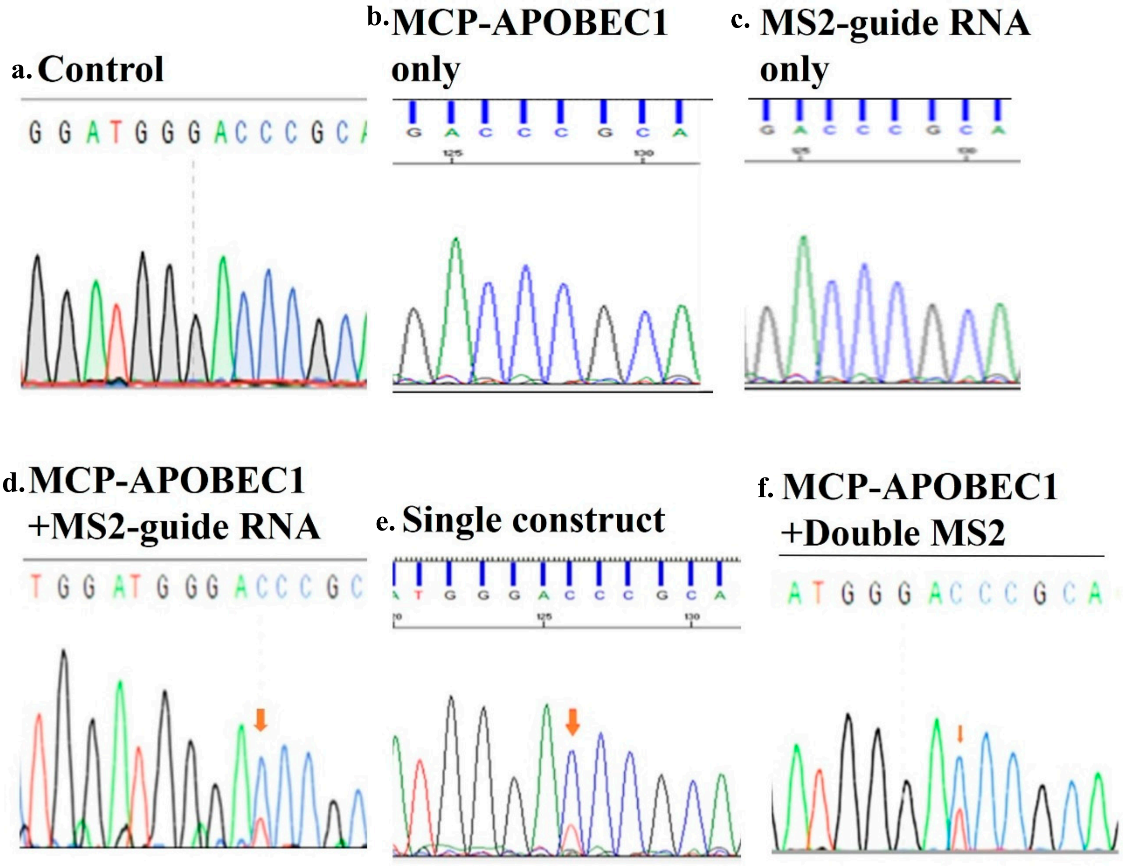

Supplementary Figure S7

Panel of sequencing result, a.: control (only fibroblast cells no transfection), b.: MCP-APOBEC1 Only, c.: MS2-guide RNA Only, d.: MCP-APOBEC1+MS2-guideRNA (Edited), e.: Single construct, and f.: MCP-APOBEC1+Double MS2 (guide RNA)
